# Painful Vater-Pacini neuroma of the digit in neurofibromatosis type 1

**DOI:** 10.3205/iprs000129

**Published:** 2019-02-08

**Authors:** Reinhard E. Friedrich, Christian Hagel

**Affiliations:** 1 Department of Oral and Craniomaxillofacial Surgery, Eppendorf University Hospital, University of Hamburg, Germany; 2 Institute of Neuropathology, Eppendorf University Hospital, University of Hamburg, Germany

**Keywords:** neurofibromatosis type 1, Vater-Pacini corpuscle, Pacinian neuroma, neurofibroma, finger

## Abstract

Vater-Pacini neuromas are rare causes of severe pain in the phalanges. The cause of this change in the tactile corpuscles is unknown. A traumatic cause has been plausibly demonstrated, at least in some cases. Here, the rare occurrence of a Vater-Pacini neuroma in a patient with neurofibromatosis type 1 is reported. The discussion addresses the difficulties of terminology and current diagnostic procedures for differentiating small nodular masses of the palm and digits. The surgical treatment leads to rapid relief of the symptoms.

## Zusammenfassung

Vater-Pacini Neurome sind seltene Ursachen heftiger Schmerzen in den Phalangen. Die Ursache dieser Veränderung der Tastkörperchen ist unbekannt. Eine traumatische Ursache ist zumindest für einige Fälle plausibel aufgezeigt worden. Das bisher sehr selten berichtete Auftreten eines Vater-Pacini-Neuroms bei einer Patientin mit Neurofibromatose Typ 1 wird berichtet. Die Diskussion geht auf die Schwierigkeiten der Terminologie und aktuelle diagnostische Verfahren zur Differenzierung nodulärer Raumforderungen der Handfläche und Finger ein. Die operative Behandlung führt zur raschen Linderung der Beschwerden.

## Introduction

Neurofibromatosis type 1 (NF1) is an autosomal dominant tumor predisposition disorder that occurs approximately once in every 2,500^th^ individual living at birth [1]. NF1 is characterized by particularly frequently detectable nerve sheath tumors. The tumors are called neurofibroma due to the admixture of reactive fibroblasts and neoplastic Schwann cells [2]. The growth of so-called cutaneous (dermal) neurofibroma is usually painless. They are not precursors of malignant tumors. Here, the often significant aesthetic disfigurements predominate the complaints of the patients. Neurofibromas and nodular plexiform neurofibroma (PNF) localized deep inside the body can be painful and are considered precancerous lesions [3]. The umbrella term “tumor predisposition disease” indicates that in NF1 patients more neoplasms occur in greater numbers than in the normal population. In addition, numerous malformations in NF1 are known, which are frequently related to dysfunctional neural structures [4].

Painful phalanges with no evidence of nodular neurofibroma are very rare in the treatment of NF1 patients [5]. This report is about a patient who was surgically explored as a result of great pain in a finger and who had an unusual histologic finding.

## Case description

### Physical investigation

**Figure 1:**
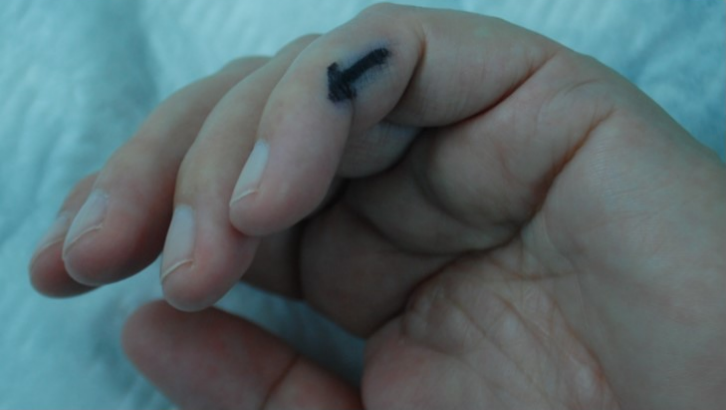
Photograph showing left small finger. Arrow indicates triggering point of painful sensation on pressure.

This 54-year-old female was referred to the Neurofibromatosis outpatient clinic of the Oral and Craniomaxillofacial Surgery Department for treatment of numerous cutaneous tumors that preferentially covered her trunk and extremities. On admission, the patient described the tumors to be painless and disfiguring. Besides the cutaneous tumors the patient sought advice for treatment of a painful region of her left small finger (Figure 1 (Fig. 1)). For some time the ulnar side of the distal phalanx of this finger had become very touch-sensitive. There was neither a tumor visible nor any other pathological alteration of the skin. However, the finger could not be examined by palpation. The suspected diagnosis was initially a glomus tumor [6] or a non-palpable nodular PNF.

### Magnetic resonance imaging

**Figure 2:**
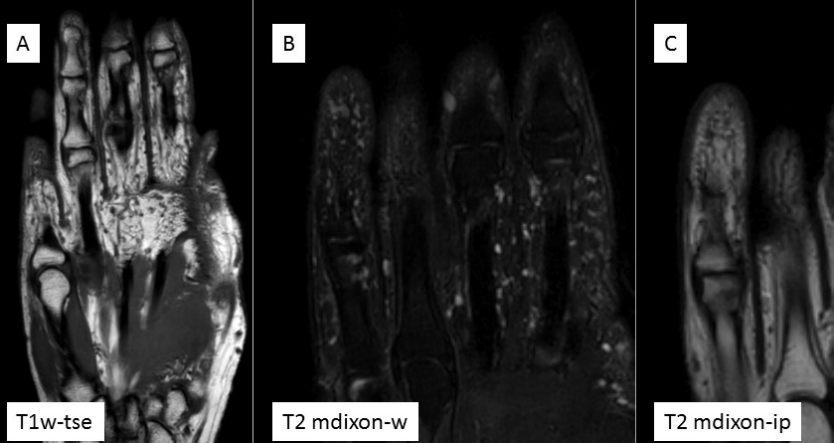
Illustration of the left hand on magnetic resonance images (MRI). The small, rounded lesions recorded in T1- and T2-weighted images were considered to be disseminated neurofibromas.

MRI at 3T revealed numerous, popcorn-like, contrast-absorbing, hyperintense formations on T2-weighted images, up to 1.2 x 1.9 cm² in size located on the palmar side of the left hand (Figure 2 (Fig. 2)). These small lesions mainly affected the metacarpal bones II and III. MRI also showed a sharply defined lesion of max. 1 cm in diameter in the subcutaneous layer at the level of the radiocarpal joint and distal to first digit that reached to the tendons of the extensor musculature. The bony structures showed a homogeneous signal. The findings were interpreted as disseminated subcutaneous and cutaneous neurofibromas of the entire left hand. Structures were found on the distal phalanx of the left small finger whose intensity pattern corresponded to that of the other lesions.

### Surgery

During surgery for the excision and vaporization of numerous neurofibromas of the trunk and extremities, the pain sensitive finger region was also explored. After incision of the skin, no tumor was visible. Therefore, a circumscribed subcutaneous excision was taken as a tissue sample and examined histologically.

### Histology

**Figure 3:**
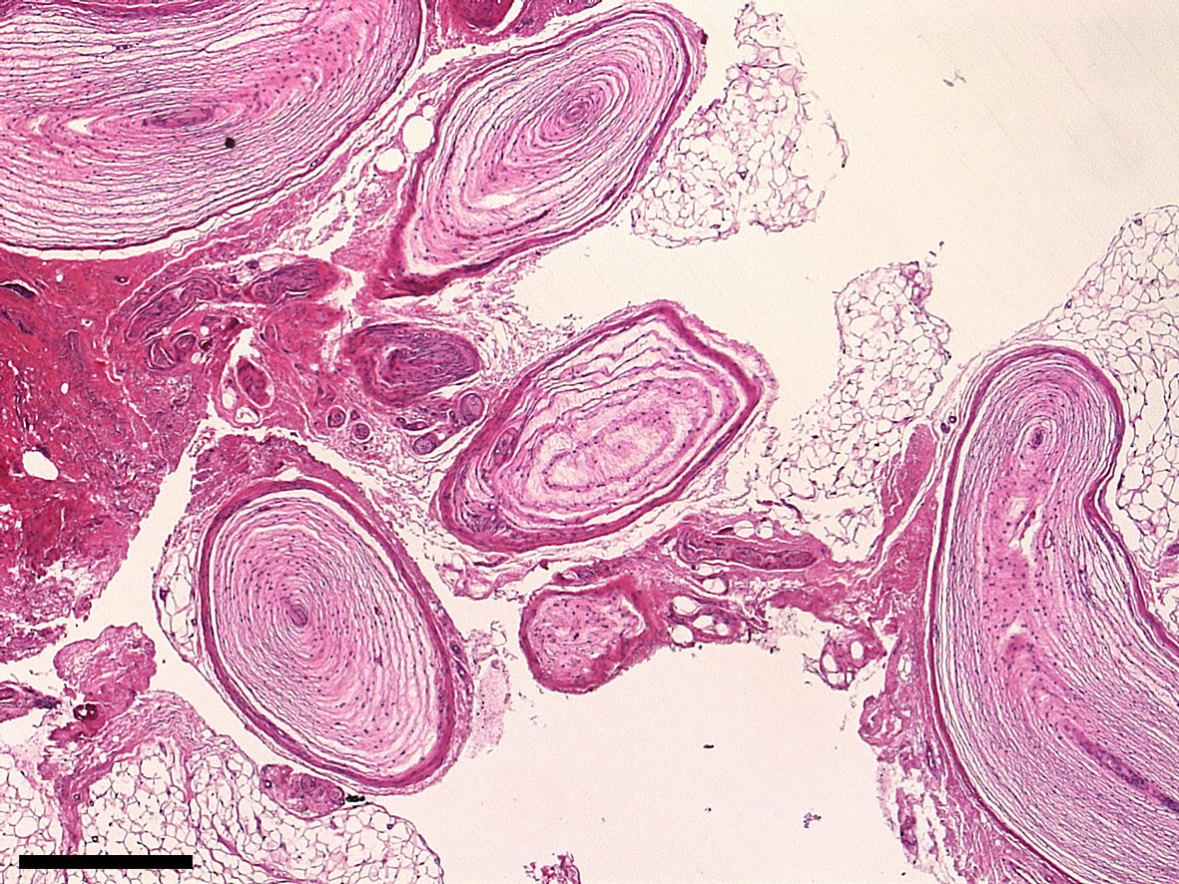
Histological appearance of Vater-Pacini neuroma type B comprising a grape like cluster of VP corpuscles embedded in adipose and connective tissue. Note that neurofibroma is present. H&E stain, scale 500 µm.

The tissue findings confirmed cutaneous neurofibromas for the numerous nodules excised from the trunk and extremities. Surprisingly, there was no evidence for a glomus tumor in the tissue sample of the finger. Smaller neurofibromas were differentiable in this tissue sample. Within the corium as well as in the subcutaneous adipose tissue there were some clustered Vater-Pacini (VP) corpuscles. Next to the corpuscles several small nerve fibers were present. Diagnosis of VP neuroma was made (Figure 3 (Fig. 3)).

### Immunohistology

The nerve fibers were positive for S100 and neurofilament. S100 immunoreactivity was especially strong in the center of the corpuscle. The perineurium of the nerve fascicles and the lamellae of the corpuscles expressed epithelial membrane antigen (EMA). 

### Follow-up

The healing process of the wounds was inconspicuous. The patient noticed that the sensations of pain had diminished significantly in the area of the small finger soon after surgery.

## Discussion

This incidental finding complements the few reports of symptomatic VP corpuscle of the hand. The peculiarity in this case is that diagnosis must be interpreted in the context of genetically-induced disease with characteristic nerve sheath tumors.

### Nomenclature

VP corpuscle (synonym Pacinian corpuscle) are tactile structures of the skin and other organs, which were named Corpuscula lamellosa by the first describer Abraham Vater (1684–1751) [7]. The same structures became again known by Filippo Pacini (1812–1883) in the medical literature, where this author delivered accurate histological findings [7]. The combination of two researchers on the same research object led to the terminological agreement that this entity bears both names. However, present medical terminology often shortens the term to Pacinian corpuscle [8], [9].

While the terminology of the physiological entity is largely maintained uniformly, designations of diseases or pathological changes of these corpuscles have been the subject of terminological difficulties. This applies in particular to the association of VP corpuscle and neurofibroma. 

A tumor originating from these subcutaneous tactile corpuscles is called (Vater-)Pacini (corpuscle) neuroma [10]. The term refers to the possibly traumatic origin of this structure [10]. VP neuroma is a rare tumor originating from the perineurial cells in a structure reminiscent of VP corpuscles. Today, VP neuroma is referred to as “extraneural perineurioma”. Such tumors are intraneurally present in larger nerves, but also in the soft tissue of the extremities, e.g. at the terminal phalanges of the fingers [11], [12]. 

### Function of VP corpuscles

VP corpuscles are rapidly adapting mechanoreceptors that respond primarily to high-frequency vibratory stimuli. These are end organs of sensory receptivity. They are not innervated by nociceptive nerve fibers. 

### Anatomy of Pacinan corpuscles

VP corpuscles are present in the dermis of various organs, such as the fingers and the palm of the hand, the conjunctiva of the eye, near joints, and other organs. The anatomical examination of hands has shown that VP corpuscles of the hand preferentially develop near portions of nerves and vessels that are in the area of the metacarpophalangeal joints and the proximal phalanx. About 300 (192–424) VP corpuscles are distributed in the hand, 60% of them are located in the digits [13]. Here, VP corpuscles are unevenly distributed: 44–60% are present in the proximal parts of the fingers, 23–48% in the metacarpophalangeal area and only 8–18% in the thenar and hypothenar region. The size of the VP corpuscles depends on the topography. VP corpuscles of the distal phalanx are smaller than receptors of the metacarpophalangeal region [13]. This difference in size of VP corpuscle has to be taken into account in the morphological analysis and evaluation of pathological findings.

### Hyperplasia of VP corpuscles

Hyperplastic VP corpuscles of the hand are rare findings [14]. A relationship between hyperplastic VP corpuscles and neurofibromatosis was considered very unlikely [15]. Hyperplastic VP corpuscles were defined by size and structure [16] using a classification of Rhode and Jennings [17]. Although this classification refers to VP neuroma, hyperplasia of the corpuscles is the defining feature of the entity [17]. Type A includes a single, enlarged corpuscle, which is attached to and beneath the epineurium of an otherwise normal-looking digital nerve. Type B includes corpuscles which are described as grape-like clusters, clumps, or aggregates of normal sized bodies. Type C is corpuscles attached to the ulnar nerve and slightly enlarged. They are located in tandem, beneath the epineurium. Corpuscles of this type act like extra branches of the nerve. Type D is hyperplastic corpuscles along the entire length of a digital nerve. Each individual or paired corpuscle is connected to the nerve by a fine, filamentous nerve fiber. Using this classification, the current findings are to be classified as Type B. 

### Pain sensation and size of the VP corpuscles

A recent review of painful VP corpuscles showed that 31 out of 36 cases of hypertrophy of the tactile corpuscles were detected [16]. Trauma as the cause of the development of Pacinian neuroma can only be safely deduced from the patient’s medical history in about half of the cases [16]. Size of the VP corpuscle is not a factor that correlates with the development of pain because VP corpuscles of normal size may also be painful [16]. This observation reduces the classification of the Pacinian neuroma according to size and arrangement to an exclusively topographic orientation. In some cases, heterotopia of the VP corpuscle is associated with the onset of pain, especially if they are in topographical proximity to the nerves [16]. However, the close relationship between VP corpuscles and the digital nerves is a normal anatomical finding and this connection is also recommended as a guiding structure of hand surgery [18].

### Simultaneous appearance of glomus tumor and VP corpuscle

In very rare cases, glomus tumors and hyperplasia of VP corpuscles may occur in the finger at the same site of pain triggering [19], [20]. None of the reports mentioned NF1 as the underlying disease of the patients. Glomus tumors are frequently detected in NF1 patients [21]. They are usually symptomatic by significant pain.

### Immunohistochemistry

In normal VP corpuscles, S100 immunostaining was recorded in selectively labelled cells resembling Schwann-cells forming the inner core [15], [22]. Glut1 immunoreactivity labelled the entire outer core [22]. EMA also labelled the outer core [15] and in another study faintly also the inner portion of the corpuscle [22]. CD34 immunoreactivity was restricted to one flat layer immediately outside the inner core. This finding was age-independent. The authors interpret this finding as indicating a functional division of the VP corpuscle, the inner neural component, and the outer non-neural component [22].

### Differential diagnosis of VP corpuscles and VP neuroma

The differential diagnosis for VP neuroma first of all includes normal VP corpuscles. Karamchandani and Rouse [23] propose certain criteria to favor a diagnosis of VP neuroma: History of local trauma, point tenderness and palpability of a nodule, surgical identification of nodule(s) related to a nerve, abnormal histology (size, number, shape, fibrosis), and postoperative resolution of pain. In the present case, only point tenderness and pain cessation are applicable to substantiate diagnosis.

### VP and MR imaging

Recently, Rhodes et al. [24] have re-evaluated MRI images of the hands of patients with inflammatory diseases. The reason for the re-evaluation was the knowledge of repeated, inexplicable “palmar dots” or “bright spots” on MRI of the hands of these patients, after surgical reports had shown that VP corpuscles were identified in the region of the conspicuous findings as seen on MRI. This finding had given rise to the assumption that VP corpuscle might have caused this radiological feature [24]. The authors point out that the radio-morphologic abnormal findings have been known to them from a number of earlier MRI studies of the hands of other patients, but until now a correlation of the “spots” with an anatomical or histological finding had not been achieved. Indeed, the distribution pattern and the size of the bright dots clearly correlated with the anatomical knowledge of the location and size of the VP corpuscle [25]. 

However, in this imaging case study, no clear distinction was made between normal and symptomatic VP corpuscles. Palpable and pain-inducing tumors were diagnosed for the separately presented cases, from which the indication for the surgical intervention had been derived.

Coincidentally, in one of the two surgically explored cases with pre-surgically performed MRI, both a VP corpuscle and a neurofibroma directly adjacent to it had been excised. NF1 had been excluded in this patient. The reproduced imaging of the MRI clearly showed the differences between the two entities according to the intensity modulation in the MRI. The neurofibroma showed a roundish-oval shape in the coronal image at T2-weighting, had a fine, almost complete hyperintense margin similar to a capsule and appeared inhomogeneously in the inner areas. The neurofibroma is many times larger than the VP corpuscles. VP corpuscles enhance in T2-weighted image, independent of whether the bodies are isolated or clustered. Findings are recorded predominantly in the palmar region. In a complementary MRI acquisition technique, the same region was further investigated using intravenous contrast-enhanced spoiled gradient-recalled echo-fat suppression (SPGRfs). Under these conditions, the neurofibroma strongly absorbed contrast medium, especially in areas previously hypointense in T2-weighted images. VP corpuscles did not enhance following SPGRfs [24]. Further morphological examination revealed the detection of a tiny internal neurofibroma in another VP corpuscle, which was not visible on the MRI even in retrospect [24]. In the publication, this finding is illustrated by a photomicrograph showing a solid tumor inside a corpuscle [24].

The radiological analysis of this report necessarily leaves open whether the excised organs or tumors had all been symptomatic. The report is a valuable extension of the assessment of small, multilocular, hyperintense space-occupying lesions on T2-weighted images of the hand. This report is interesting for the assessment of the presented findings for several reasons. First, at least in one case, the neurofibroma was apparently the trigger for the reported pain. At the very least, imaging is likely to favor this association because the neurofibroma is significantly larger than the neighboring VP corpuscle. In fact, the report explicitly notes the palpation of a tumor as the trigger point of the pain sensations. This tumor had emerged superficially to the proximal phalanx of the long finger. Illustrative MRI images contain the neurofibroma at this point. In addition, this recent report reiterates that there is a threshold of detection for the identification of small neurofibroma (and small VP corpuscles) in MRI. On the other hand, the study shows that VP corpuscle and neurofibroma, from an unknown size, have different signal behavior that can be used diagnostically. The case presented is probably a coincidental finding of two entities, the VP corpuscle in disseminated neurofibromatosis. VP corpuscle contain Schwann cells and these may thus be the origin of neurofibromas. At least the development of one neurofibroma within a VP corpuscle is convincingly demonstrated [24], whereby the lamellae are thinned out by the central proliferative tumor. Further technical details about the used MRI device are not given in the authors’ statement.

### VP corpuscle, VP neuroma and neurofibroma

In neurofibromas, occasionally organoid structures are recognized reminiscent of “Vater-Pacini lamellar bodies” [26], [27], another synonym for VP corpuscles. The formation of VP corpuscle-like structures in neurofibroma has led to the introduction of the term “Pacinian neurofibroma” [28]. This designation is currently considered a misnomer [26], but still in use [16], [29], [30], [31], [32], [33]. The delineation of neurofibroma from pathological changes defining VP corpuscles was shown as an example of a painless mass of the distal phalanx of a finger that proved to be a neurofibroma with organoid features. The organoid features resembled VP corpuscles [26]. The occasionally striking similarity of a neurofibroma with the tactile corpuscle appears to be due to the arrangement of nucleus-containing and nuclear-free sections in the histological sections [26]. The peculiar morphological characteristics of the plexiform neurofibroma (“Rankenneurom”) in that case were the predominantly focal proliferation of the perineurial connective tissue. This proliferation can give rise to pear-shaped distensions of nerve endings, reminiscent of Vater-Pacini lamellar bodies, but otherwise unrelated to these tactile bodies [26]. Interestingly, already Altmeyer pointed to the fact that there was no relation between organoid neurofibroma resembling VP corpuscle and NF1 in his review of the literature at that time [26]. 

### VP corpuscle in NF1

Schochet and Barrett [29] report on a young patient who developed neurofibroma of the hand with aberrant tactile corpuscle. The tumor was first noticed and surgically treated in the 7-week-old child. However, the tumor recurred and was excised a second time. The second procedure lasted permanent relief of the painless tumor. The authors diagnosed von Recklinghausen’s disease, because the patient had numerous *café-au-lait* spots all over the body. The hand tumor showed the differentiation of a VP corpuscle with central parts of a neurofibroma. The authors consider as a cause of this differentiation disorder either a primary neurofibroma-driven, dysmorphic development of the tactile corpuscle or the ingrowth of a neurofibroma into the already formed VP corpuscle. 

Yan et al. [5] reported on a symptomatic, hyperplastic VP corpuscle in a NF1 patient. The individual had both pain and sensory disturbances in the affected ring finger area. The surgical exploration was carried out on suspicion that a neurofibroma was the cause of the symptoms. However, on the MRI, there was no mass in the hand. There was a significant improvement in the neurological findings after the surgical procedure. The histological investigation revealed up to 12 VP corpuscles localized in the subcutaneous adipose tissue. The structure of the corpuscle consisted of concentric lamellae of the connective tissue, which encircled a central axis. The long axis of the corpuscle was up to 3.5 mm long. The maximum number of lamellae was 35. In the immunohistochemical study of the specimen, strong immunoreactivity to the S100 protein was diagnosed in the central axis. The capsule was positive for the detection of EMA. Throughout the work, the authors use the term “hypertrophy” of the VP corpuscle and differentiate their findings from that of Pacinian neurofibroma. For the latter, they would have expected a corpuscle-like differentiation with myxoid stroma.

## Conclusions

The rare finding of symptomatic Pacinian neuroma also occurs in patients with NF1. Isolated reports attest to the close topographical relationship between neurofibroma and a deformed VP corpuscle. Differential diagnosis of the neuroma to (heterotopic) VP corpuscle can be demanding. The painfulness of neuromas reported in the individual findings also applies to patients with NF1. The excision of the lesion is an effective therapy.

## Notes

### Competing interests

The authors declare that they have no competing interests.

### Funding

This research did not receive any specific grant from funding agencies in the public, commercial, or not-for-profit sectors.
